# ECG-based estimation of dispersion of APD restitution as a tool to stratify sotalol-induced arrhythmic risk

**DOI:** 10.1016/j.jelectrocard.2015.06.006

**Published:** 2015

**Authors:** A. Mincholé, A. Bueno-Orovio, P. Laguna, E. Pueyo, B. Rodriguez

**Affiliations:** a Department of Computer Science, University of Oxford, Oxford, United Kingdom; b Biomedical Research Networking Center in Bioengineering, Biomaterials and Nanomedicine (CIBER-BBN), Spain; c BSICoS Group, Aragón Institute of Engineering Research (I3A), IIS Aragón, Universidad de Zaragoza, Zaragoza, Spain

**Keywords:** Rate adaptation, APD restitution, cardiotoxicity, sotalol

## Abstract

**Background:**

Increased spatial dispersion of restitution properties has been associated to arrhythmic risk. An ECG-based index quantifying restitution dispersion, DRest, is evaluated in patients who experienced Torsades de Pointes (TdP) under sotalol challenge and compared with the response in healthy subjects.

**Methods and Results:**

ECG recordings were analyzed for quantification of DRest and QTc, among others biomarkers. DRest provides improved discrimination following sotalol administration between TdP and healthy subjects ([min–max]: [0.18–0.22] vs [0.02–0.12]), compared to other biomarkers including QTc ([436–548 ms] vs [376–467 ms]). Results in healthy subjects are in agreement with simulations of sotalol effects on a human tissue electrophysiological model.

**Conclusions:**

This case study supports the potential of DRest for improved arrhythmia risk stratification even with QTc values below 450 ms.

## Highlights

• Several rate-dependent ECG-based biomarkers were evaluated in three patients who develop Torsades des Pointes after sotalol intake and in twenty five healthy volunteers who do not.
• Dispersion of restitution quantified from the ECG, DRest, identified better drug-induced cardiotoxicity than the conventionally used QTc.
• Neither DRest nor QTc shows sotalol-induced changes in subjects who do not develop TdP.
• Computer simulations corroborate the results of the ECG analysis and suggest that DRest is able to quantify the arrhythmogenic substrate in patients at risk of developing drug-induced arrhythmias.

## Introduction

Pharmacological treatment is often administered to patients at arrhythmic risk through the use of class III anti-arrhythmic drugs. However, in patients with a previous arrhythmogenic substrate, class III drug-induced electrophysiological alterations can sometimes worsen or even induce new arrhythmias, such as Torsades de Pointes (TdP) [1]. Risk stratification in patients undergoing anti-arrhythmic pharmacological treatment is often performed using the surface electrocardiogram (ECG) [2]. Within this scope, the development of specific and selective ECG-based biomarkers for arrhythmic risk stratification is therefore critical.

Class III anti-arrhythmic drugs, such as sotalol, block the rapid component of the delayed rectifier current (I_Kr_), leading to action potential duration (APD) prolongation at the cellular level and the resulting QT prolongation at the ECG [3]. QT prolongation is the main biomarker used to assess drug cardiotoxicity [2], although its main limitation is its low specificity: neither all drugs that prolong the QT interval result in arrhythmia, nor all pro-arrhythmic drugs prolong the QT interval [2,4–6], even though for the particular case of TdP antiarrhythmic drugs many of them show QTc prolongation.

A large body of research has been devoted to the development of novel ECG-based biomarkers for arrhythmic risk stratification. Some studies have suggested that the adaptation of the QT interval to a sudden change in heart rate can be used as a clinical arrhythmic risk marker [7]. Moreover, prolongation of the T-peak to T-end (T_pe_) interval on the ECG, which aims to quantify ventricular dispersion of repolarization [3], has been associated with an increased risk of sudden cardiac death [8], although one study showed shorter T_pe_ in cardiovascular patients dying from any cause compared to the ones who survived [9]. However, the potential of these biomarkers in risk stratification under sotalol challenge is unknown.

An additional pro-arrhythmic factor of sotalol is its reverse use dependence, which modifies APD restitution properties at the cellular level, increasing the slope of the APD restitution curve [10]. The dynamic APD restitution curve quantifies the stationary relationship between the APD and the pacing cycle length (RR interval) at different RR values. This sotalol-induced steeper APD restitution slope may lead to an increased spatial dispersion of restitution properties, which has been proposed to act as a potent arrhythmogenic substrate, associated with the inducibility of ventricular arrhythmias [11]. Recently, the spatial dispersion of APD restitution was successfully quantified from the ECG by a novel index, DRest, which accounts for the rate normalized differences of the T_pe_ interval under different stationary conditions [12]. This biomarker has also been used in previous studies and proposed as an independent predictor of sudden cardiac death in patients with chronic heart failure [13]. DRest has also been considered for improved discrimination of patients with hypertrophic cardiomyopathy, a genetic disease and main cause of sudden cardiac death in patients under 35 years old [14].

The aim of this case study is to provide new evidence of the potential of novel ECG-based biomarkers, such as DRest and rate adaptation of QT and T_pe_ intervals, to improve the stratification of patients at risk of developing drug-induced arrhythmias. We evaluated the power of novel and established ECG-based biomarkers to discriminate three patients who developed TdP under sotalol challenge, with respect to healthy volunteers who did not develop arrhythmic events.

Additionally, a computer simulation study was performed to investigate the effect of sotalol-induced I_Kr_ block on APD restitution dispersion. The *in silico* results indicate that I_Kr_ block does not significantly increase DRest in healthy human tissue, therefore suggesting that the increased pro-arrhythmic risk of sotalol in patients developing TdP is likely to be caused in synergy with their disease-related cardiac substrate.

## Methods

### Data

Cardiotoxic effects of sotalol intake were analyzed in two distinct groups consisting of 3 TdP patients and 25 healthy volunteers. Due to ethical and practical constrains, data obtained in drug-induced TdP patients are scarce but very valuable, and they were obtained from the ECG database available in the framework of the THEW project (University of Rochester, ID:E-OTH-12-0006-009). The TdP patients had a previous history of TdP and were enrolled for a diagnostic test based on intravenous dl-sotalol IV to unmask latent repolarization abnormalities. They experienced drug-induced TdP after sotalol intake (2 mg/kg body weight). To determine the ability of ECG-based biomarkers for arrhythmic risk stratification, a subset of a second database conducted at the Pharmacia's Clinical Research Unit [15] was considered, consisting of 25 recordings from healthy volunteers that were made available to us for this study. This subset selection was based on availability of the recordings for this particular study. The rest of the recordings in the original database were not available for analysis, but the 25 records were considered sufficient to be paired with the just 3 cases of TdP patients. None of these healthy subjects developed any arrhythmic episodes after sotalol challenge with 160 mg of oral sotalol [15]. Seven of the healthy subjects, who did not experience prolongation of the QTc interval over 450 ms after the first dose, were administered a second dose of 320 mg and hereinafter will be referred to as “Healthy + second sotalol dose”. The intravenous and oral doses used in our study are equivalent based on the doses regimen approved by the US Food and Drug Administration (FDA), which suggest the replacement of 75 mg intravenous sotalol by 80 mg oral sotalol [16]. Taking into account that the mean weight of healthy volunteers was 74 kg [15], the intravenous dose for 160 mg oral sotalol was 2.02 mg/kg body weight, which is equivalent to the dose administered to the TdP patients. Moreover, at equivalent concentrations as considered in this study, intravenous and oral sotalol have been shown to yield similar QT and QTc effects [17].

A 12-lead ECG recording at a sampling frequency of 180 Hz was obtained from each subject. The first hour excerpt was used for the computation of biomarkers, using the lead with the highest signal-to-noise ratio. Other leads were also analyzed, with no influence on the obtained results as demonstrated in this work. ECG recordings were available from healthy volunteers before and after drug intake, but only after drug intake for TdP patients due to the original definition of the clinical study.

### ECG-based biomarkers

**Fig. 1:**
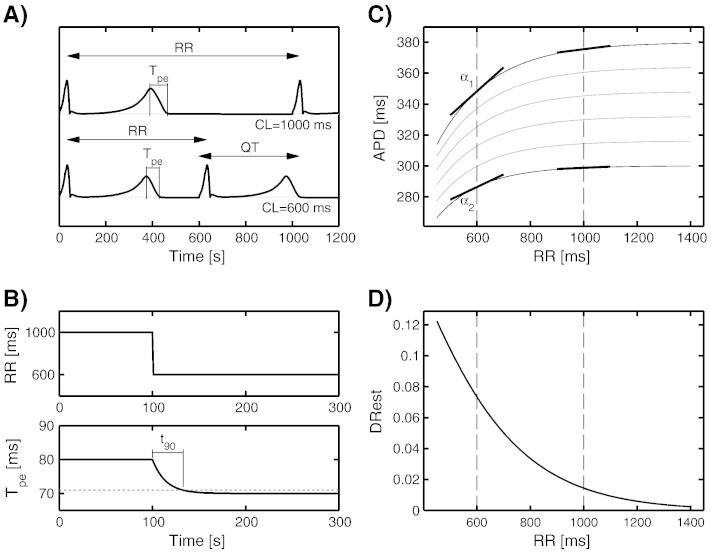
(A) Simulated ECGs for two RR intervals or cycle lengths (CLs) of 1000 and 600 ms under stationary conditions. (B) Response of the T_pe_ interval after a change in the RR interval from 1000 to 600 ms, and illustration of *t*_90_^Tpe^ computation. (C) Restitution curves corresponding to different spatial locations in the heart. Each curve represents the APD at different stationary RR intervals. *α*_1_ and *α*_2_ represent the maximum and minimum slopes for RR = 600 ms. (D) Difference between maximum and minimum restitution slopes, DRest, computed for a range of RR intervals.

Fig. 1 illustrates the ECG biomarkers quantified in this study. Fig. 1A shows representative simulated ECG signals under stationary conditions for RR intervals of 1000 and 600 ms. The adaptation of the T_pe_ interval after a sudden change of RR intervals from 1000 to 600 ms is shown in Fig. 1B. Fig. 1C illustrates differences in dynamic restitution curves due to spatial heterogeneity in repolarization in the human ventricles. Each curve represents the stationary APD at one ventricular location for different stationary RR intervals. *α*_1_ and *α*_2_ denote the maximum and minimum dynamic restitution slopes at a given RR interval. The spatial dispersion in APD restitution slopes, DRest, is dependent on the specific RR interval, as illustrated in Fig. 1D.

The following ECG biomarkers for drug-induced risk stratification were computed:

- QTc (Fig. 1A): QT interval corrected by the effect of heart rate, using Fridericia's formula (classical biomarker to assess cardiotoxicity).
- QT and T_pe_ rate adaptation *t*_90_^QT^ and *t*_90_^Tpe^ (Fig. 1B): time to complete 90% of the total adaptation of QT and T_pe_ intervals (respectively) after an abrupt change in heart rate [7].
- DRest (Fig. 1D): spatial dispersion of APD restitution slopes as illustrated in Fig. 1C. The DRest biomarker (proposed and referred to as ∆*α* in Ref. [12]) represents the ratio:
  (1) $$\mathrm{DRest}=\frac{\Delta T_{pe}}{\Delta RR}$$
  where ΔT_pe_ is the T_pe_ difference at two different RR intervals [12]. Due to the natural beat-to-beat variability in RR intervals, Eq. (1) is estimated with the methodology described in [18] to avoid the need of stationary RR segments. Therefore, in order to properly compute DRest, long periods of time of at least 10–15 minutes with heart rate changes present are needed.

### Simulation of sotalol-induced changes in dispersion of APD restitution

An *in silico* simulation study was conducted to simulate the effect of sotalol-induced I_kr_ block on dispersion of repolarization in healthy human ventricular substrates. Propagation of electrical excitation was simulated through a transmural ventricular slice of human tissue, as described in Ref. [12]. The well-established human ten Tusscher–Panfilov action potential model [19], which provides a good characterization of human APD restitution dynamics, was used to represent membrane dynamics. The stimulation protocol, activation sequence and transmural heterogeneities were defined as reported in Ref. [12]. The effects of sotalol were simulated for an IC50 dose, resulting in a 50% reduction in I_Kr_ conductance.

Spatial dispersion of APD restitution was quantified in the simulations by computing the derivative of the curve that relates each stationary T_pe_ with respect to the corresponding stationary RR. The range of simulated RR intervals was from 500 to 1500 ms.

## Results

### Risk stratification of ECG-based biomarkers

**Fig. 2:**
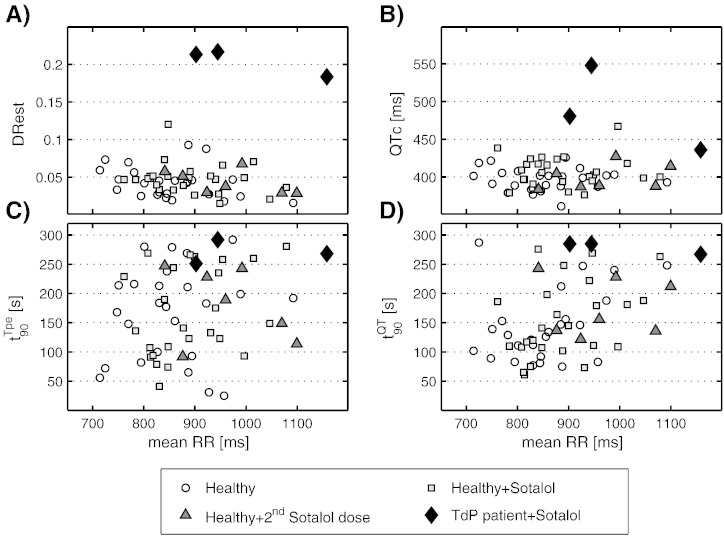
Quantification of ECG-based biomarkers for TdP patients after sotalol challenge (filled diamonds), and healthy volunteers before (open circles), after sotalol administration (gray squares) and after the second sotalol dose (filled triangles). (A and B) Rate adaptation biomarkers of T_pe_ and QT intervals (*t*_90_^Tpe^ and *t*_90_^QT^, respectively). (C) Results corresponding to the biomarker DRest, quantifying dispersion of APD restitution. (D) Results for the QTc interval.

Fig. 2 presents the different ECG-based biomarkers quantified in this case study, plotted against the mean RR interval for each subject. Results are provided after sotalol administration for TdP patients, and before and after sotalol intake for healthy volunteers. DRest shows the best discrimination power between both population groups (Fig. 2A). These results highlight that TdP patients exhibit much larger DRest values than any of the healthy subjects, either before or after equivalent sotalol intake. Significant differences between the healthy group with the first dose and the TdP groups after sotalol intake were observed for DRest with a *p*-value of 0.006 when the non-parametric Mann–Whitney U test was performed. Importantly, 160 mg sotalol administration does not significantly increase dispersion of APD restitution DRest in the healthy subgroup (*p*-value = 0.27). Moreover, DRest for all TdP patients lay beyond six standard deviations of the mean DRest value of healthy volunteers.

For comparison purposes, data for the commonly used biomarker of drug cardiotoxicity QTc are also presented (Fig. 2B). Sotalol administration (160 mg) only led to a slight increase in QTc (about 10 ms in mean) in the healthy group. Although 2 TdP patients showed marked QTc prolongation, the third TdP patient exhibited a similar QTc as those in the non-inducible TdP healthy cohort. When compared to DRest, less significant differences were found (*p*-value = 0.009) when the Mann–Whitney U test was performed.

When performing other standard tests as the Student's t test (assuming unequal variances and unequal sample size) to evaluate the differences in DRest and QTc between TdP and healthy volunteers (160 mg sotalol administration), statistically significant differences were observed in DRest (*p*-value = 0.0013), while for QTc, the *p*-value was 0.1318. Similar results were obtained when applying other QT correction formulas, such as Bazett's, which also resulted in QTc failing to provide a complete separation between groups.

The results for the time to complete 90% rate adaptation of the T_pe_ (*t*_90_^Tpe^) and QT (*t*_90_^QT^) intervals are shown in Fig. 2C and D. A large variability is observed within the healthy group in terms of rate adaptation times (both before and after 160 mg sotalol intake), with differences over 150 and 100 s for T_pe_ and QT adaptation, respectively. The rate adaptation biomarkers show values for TdP patients within the upper range obtained for healthy subjects.

Regarding the second 320 mg sotalol dose given to seven healthy subjects, results on DRest, QTc, and *t*_90_ values of T_pe_ and QT adaptation are in the same range as for the previous 160 mg dose in healthy subjects. This is further supported in Table 1, which summarizes the results presented in Fig. 2 for the different ECG-based biomarkers as well as T_pe_c, representing the T_pe_ interval corrected by the effect of heart rate using Fridericia's formula. Results are presented as 20 and 80 percentiles bounds in the healthy and healthy + sotalol groups to avoid possible outliers, the full range for healthy subjects + second sotalol dose, and individual values for the three TdP patients. We cannot detect electrophysiological abnormalities in the TdP patients in time interval-based biomarkers such as T_pe_ and RR intervals, as they present values within normal ranges and no significant differences were found.

**Fig. 3:**
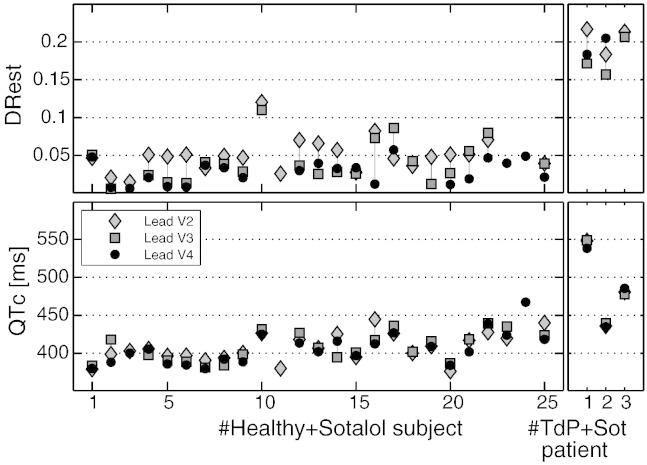
Sensitivity of DRest and QTc biomarkers with respect to V2–V4 leads. Biomarker values for the 25 healthy subjects after sotalol administration and the 3 drug-induced TdP patients are shown for leads V2, V3 and V4 (if delineation can be successfully performed).

*t*_90_, QTc and DRest were computed using the ECG lead with the highest signal-to-noise ratio, which in this study are V2, V3 or V4. In order to evaluate whether these results depend on the lead selection, Fig. 3 shows the values of the biomarkers DRest and QTc for leads V2 to V4 (the ones that have better delineation of the T_pe_ interval). This figure shows that our results are consistent across leads and therefore that the lead selection does not affect the main conclusions of this study.

### Role of I_Kr_ block in determining sotalol-induced changes in APD restitution dispersion

Computer simulations were conducted to investigate the effect of sotalol-induced I_Kr_ block in the biomarker DRest under non-diseased conditions. The aim was to help in the interpretation of the results obtained for healthy volunteers, by identifying effects solely ascribable to I_Kr_ block rather than to the presence of an additional cardiac disease-related substrate.

**Fig. 4:**
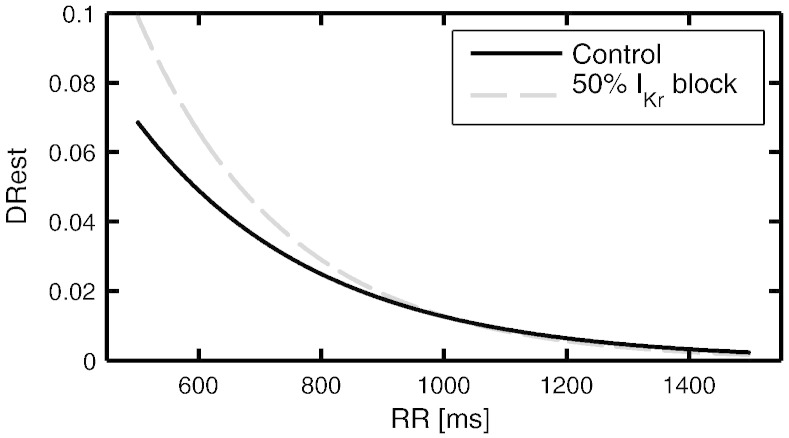
Simulated DRest measured in a human 2D tissue preparation in control (black, solid line) and under 50% I_Kr_ block (gray, dashed line), mimicking the effect of sotalol.

Fig. 4 presents the simulation results for the biomarker DRest in control conditions (blue solid line) and for 50% I_Kr_ block (red dashed line). The *in silico* results support that I_Kr_ block can actively modulate the pro-arrhythmic substrate, increasing APD restitution dispersion at fast pacings. However, these differences are hardly noticeable for RR intervals above 800 ms. The simulation results are hence in agreement with the ECG analysis presented in Fig. 2A, showing similar values in DRest before and after sotalol administration for healthy subjects, as most of them presented RR intervals above 800 ms.

## Discussion

In this study, we evaluated the potential of novel and established rate dependent ECG-based biomarkers for the stratification of 3 TdP patients against a cohort of healthy volunteers following sotalol administration. For the three patients who developed TdP after sotalol administration, increased APD restitution dispersion (quantified by DRest) was observed and shown to provide the best discrimination with respect to the healthy group, compared to other biomarkers including QTc. Computer simulation results show agreement with the observations of DRest in healthy volunteers, and they suggest that I_Kr_ block *per se* does not cause alterations of DRest in a healthy substrate.

Two different rate dependencies have been considered in this study. Firstly, a “short time” rate dependency or adaptation, which is in the very core definition of some of the analysed biomarkers. Thus, *t*_90_ values represent the rate adaptations of the QT and T_pe_ intervals to changes of heart rate, as computed in reference [18]. Rate adaptation of T_pe_ is also used in the computation of DRest to remove the memory effect between T_pe_ and RR intervals. This memory compensation is described in detail in Ref. [18]. Secondly, another “long term” rate dependency was considered by representing biomarkers against mean RR values of each analysed ECG excerpt. In normal conditions DRest is higher for shorter RR intervals [12]. In this study, as the RR intervals of the subjects are between 750 and 1100 ms, differences between DRest values are expected to be around 0.02 (as evidenced in Ref. [12] and in simulations shown in Fig. 4). Therefore, the larger differences in DRest found between healthy subjects and TdP patients (around 0.15), are not accounted for the RR dependency or the inter subject variability for healthy volunteers.

### ECG quantification of APD restitution dispersion

Healthy subjects did not present differences in DRest after sotalol intake, whereas all patients developing sotalol-induced TdP exhibited substantially higher DRest values compared to the healthy group.

Large restitution gradients (DRest ~ 0.2) were observed in the 3 patients developing TdP under sotalol challenge. This is in agreement with previous studies showing correlation between increased APD restitution dispersion and a higher inducibility of ventricular tachyarrhythmias [11]. These values are in fact considerably larger than those shown in healthy volunteers in our study and also previously reported DRest magnitudes in other cohorts of patients at high arrhythmic risk in the absence of sotalol. A recent study considering ECG recordings from patients with chronic heart failure has reported DRest values from non-sudden cardiac death victims of 0.026 ± 0.003 and from SCD victims of 0.052 ± 0.013 [13]. An additional study on hypertrophic cardiomyopathy patients [14] has reported DRest values of around 0.065. The remarkable differences with the sotalol-induced TdP patients analyzed in this study suggest an active modulation by sotalol of the underlying pro-arrhythmic substrate, which can be quantified from the body-surface ECG by means of the DRest biomarker. Therefore, the DRest values found in patients with a pro-arrhythmic substrate in previous studies (in the absence of sotalol) are substantially smaller than those obtained in the TdP patients following sotalol administration in our case study (DRest of around 0.2).

Simulation results presented in Fig. 4 indicate that I_Kr_ block does not significantly increase DRest in healthy human tissue, in agreement with the ECG analysis results obtained from the healthy subjects. Indeed, when increasing the dose to 320 mg oral sotalol, DRest values did not alter with respect to before and after 160 mg oral sotalol intake. These results suggest that the increased pro-arrhythmic risk of sotalol in patients developing TdP is likely to be caused by synergistic effects of sotalol and their disease-related cardiac substrate.

### Rate adaptation of the QT and T_pe_ intervals

Within the group of healthy volunteers who did not develop arrhythmias after sotalol administration, rate adaptation of the QT and T_pe_ intervals was not significantly affected by sotalol administration (see Fig. 2C and D). However, a prominent variability was observed in these biomarkers, over 100 s in the healthy group (see Table 1). This variability may be due to the dependence of rate adaptation on the initial RR interval and the change in RR interval, which are different for each ECG recording.

For the three patients who developed TdP, rate adaptation times *t*_90_ for QT and T_pe_ show moderately high values, although in the range of the healthy group. Previous studies have shown a more clear association between slow rate adaptation of repolarization and a higher propensity of suffering life-threatening arrhythmias [7].

### Limitations and further studies

We present the results of this study as a case report, given the small number of available data for patients developing TdP after sotalol administration. The absence of ECG recordings prior to drug intake in these patients and the lack of a common disease condition in this group, also hamper our ability to ascertain whether DRest represents a measure of the baseline pro-arrhythmic substrate or this substrate develops dynamically after I_Kr_ blockade. In spite of these limitations, this case study provides additional evidence on the potential of DRest for arrhythmic risk stratification by demonstrating an improved performance for discrimination with respect to the classical QTc, and supporting the need to evaluate the novel biomarker in further studies. Computing DRest requires the presence of heart rate changes over long periods of time. This represents an additional requirement with respect to other biomarkers such as QTc, and the acquisition of ECG recordings needs to be designed appropriately to be able to compute DRest. To the best of our knowledge, additional recordings are not easily accessible at present, and therefore we present our findings in the form of a case report supporting DRest as a potentially useful biomarker to take into consideration in future studies.

The evidence presented in this case study therefore encourages the design of future studies including long ECG recordings in the presence of heart rate changes, from patients treated with sotalol or patients with a previous history of TdP, who do and do not develop TdP under pharmacological challenge.

## Conclusion

In summary, the present case study presents evidence for restitution dispersion, evaluated from the ECG using the index DRest, as a promising biomarker for pro-arrhythmia stratification. In this dataset, DRest exhibited clear discrimination between TdP patients and healthy volunteers, even for the TdP patient with a normal QTc value of 436 ms. This suggests that DRest could be used to identify patients at high risk of developing TdP, even with a normal QTc. The results therefore point toward a promising potential for DRest, worth exploring in larger studies to allow for a more thorough comparison with other biomarkers.

## Figures and Tables

**Table 1:** [20 Percentile–80 percentile] bounds for the different ECG biomarkers in healthy subjects before and after sotalol intake, whole range of healthy subjects after second sotalol dose, and values for the three sotalol-induced TdP patients (TdP1, TdP2 and TdP3).

	RR [ms]	DRest	QTc [ms]	*t*_90_^Tpe^ [s]	*t*_90_^QT^ [s]	T_pe_c [ms]
Healthy	776–925	0.025–0.058	384–407	77–227	86–200	82–98
Healthy + Sotalol	815.8–951	0.033–0.054	394–424	93–259	104–210	83–96
Healthy + 2nd dose	840–1100	0.029–0.068	384–427	92–247	122–243	69–102
TdP1 + Sotalol	944.6	0.22	548	292	285	116
TdP2 + Sotalol	1158.1	0.18	436	268	267	77
TdP3 + Sotalol	902.3	0.21	480	251	285	107

Biomarkers analyzed were dispersion of APD restitution (DRest), QTc, the rate adaptation times of QT and T_pe_ intervals (*t*_90_^Tpe^ and *t*_90_^QT^) and T_pe_c.
